# Main ecological drivers of woody plant species richness recovery in secondary forests in China

**DOI:** 10.1038/s41598-018-35963-7

**Published:** 2019-01-22

**Authors:** Xiaofei Liu, John Garcia-Ulloa, Tina Cornioley, Xuehua Liu, Zhiheng Wang, Claude Garcia

**Affiliations:** 1 State Key Joint Laboratory of Environmental Simulation and Pollution Control, and School of Environment, Tsinghua University, Beijing, 100084 China; 2 Institute of Terrestrial Ecosystems, Department of Environmental Systems Science, Swiss Federal Institute of Technology Zurich (ETH Zurich), Zurich, 8092 Switzerland; 3 Department of Ecology and Key Laboratory for Earth Surface Processes of the Ministry of Education, College of Urban and Environmental Sciences, Peking University, Beijing, 100871 China; 4 Research Unit Forests and Societies, Centre International de Recherche Agronomique pour le Développement (CIRAD), Montpellier, 34392 France

## Abstract

Identifying drivers behind biodiversity recovery is critical to promote efficient ecological restoration. Yet to date, for secondary forests in China there is a considerable uncertainty concerning the ecological drivers that affect plant diversity recovery. Following up on a previous published meta-analysis on the patterns of species recovery across the country, here we further incorporate data on the logging history, climate, forest landscape and forest attribute to conduct a nationwide analysis of the main drivers influencing the recovery of woody plant species richness in secondary forests. Results showed that regional species pool exerted a positive effect on the recovery ratio of species richness and this effect was stronger in selective cutting forests than that in clear cutting forests. We also found that temperature had a negative effect, and the shape complexity of forest patches as well as the percentage of forest cover in the landscape had positive effects on the recovery ratio of species richness. Our study provides basic information on recovery and resilience analyses of secondary forests in China.

## Introduction

The future of forests in the Anthropocene depends on balance between forest recovery and human-driven deforestation^1^. Resilience, the capacity of an ecosystem to recover to pre-disturbance states from a disturbance, is key for forest restoration^2^, biodiversity conservation^3^ and maintenance of ecosystem services^4^. Much effort has been conducted for understanding the recovery patterns of forest biomass^5^, species composition^6,7^, species diversity and functional diversity^8^. Identifying ecological drivers that influence these patterns is of particular importance^9^, given the difficulties to anticipate the outcomes of forest recovery^10,11^. A better understanding of these drivers could promote more cost-effective policies and management as well as more comprehensive ecological impact assessments^12–14^.

China holds a large range of globally important forest ecosystems across a wide range of climates in both tropical and temperate regions^15^. Over the last 20 years, China has implemented numerous national policies for forest protection in order to slow down the forest degradation in association with the rapid economic growth^16^. The Natural Forest Protection Program promotes the conservation of natural forests through logging bans^17^ and the Nature Reserves Construction Program has been key in establishing nature reserve networks in the country^16^. Similar to other regions elsewhere^18^, the naturally regrowing forests which we call secondary forests in this paper, are becoming more and more important in China because they are potential reservoirs for primary forest biodiversity and sources of essential ecosystem functions^9,19^. At present, secondary forests account for 57% of the total forest resources in China^20^ and forest biodiversity has increased, which thanks to these progressive forest protection policies^16^.

Although China has a large area of secondary forests and the biodiversity is gradually recovering, the ecological drivers influencing the performance of natural forest recovery are poorly understood^21^. The recovery of secondary forests is affected by various drivers operating at multiple spatio-temporal scales^9^. Studies have shown that logging history, climatic conditions, land use and forest attributes can all affect forest recovery^5,22–25^. A global meta-analysis showed that forest recovery was more successful when previous logging was less intensive and the landscape was less fragmented^22^. In the Neotropics, the biomass recovery in secondary forests increased with the increase in rainfall^5^. During secondary succession, species availability (e.g. high efficient propagation of seeds) is crucial to whether the secondary forests can recover to primary forest states^7,19^. Regional species pool is a set of species occurring in a particular region that can potentially inhabit a site because of suitable local ecological conditions^26^. Larger regional species pool implies that there are more species that are able to grow in this specific ecological conditions, and therefore to have better recovery performance after disturbances^11^. However the relative contribution of these ecological drivers that influence the recovery of secondary forests in China still need to be explored. Identifying these drivers is critical for resilience and secondary succession analyses as well as for advancing sustainable management of forests in future.

A previous published meta-analysis based on 125 pairs of secondary-primary forest data across China has revealed the recovery patterns of woody plant species richness in secondary forests^24^. Here, following up on this previous meta-analysis, we further incorporate data on the logging history, climate, forest landscape and forest attribute to identify the main ecological drivers that influence the observed natural recovery of woody plant species richness in secondary forests in China. We hope this study could provide basic information on recovery and resilience analyses of secondary forests in China.

## Results

**Figure 1:**
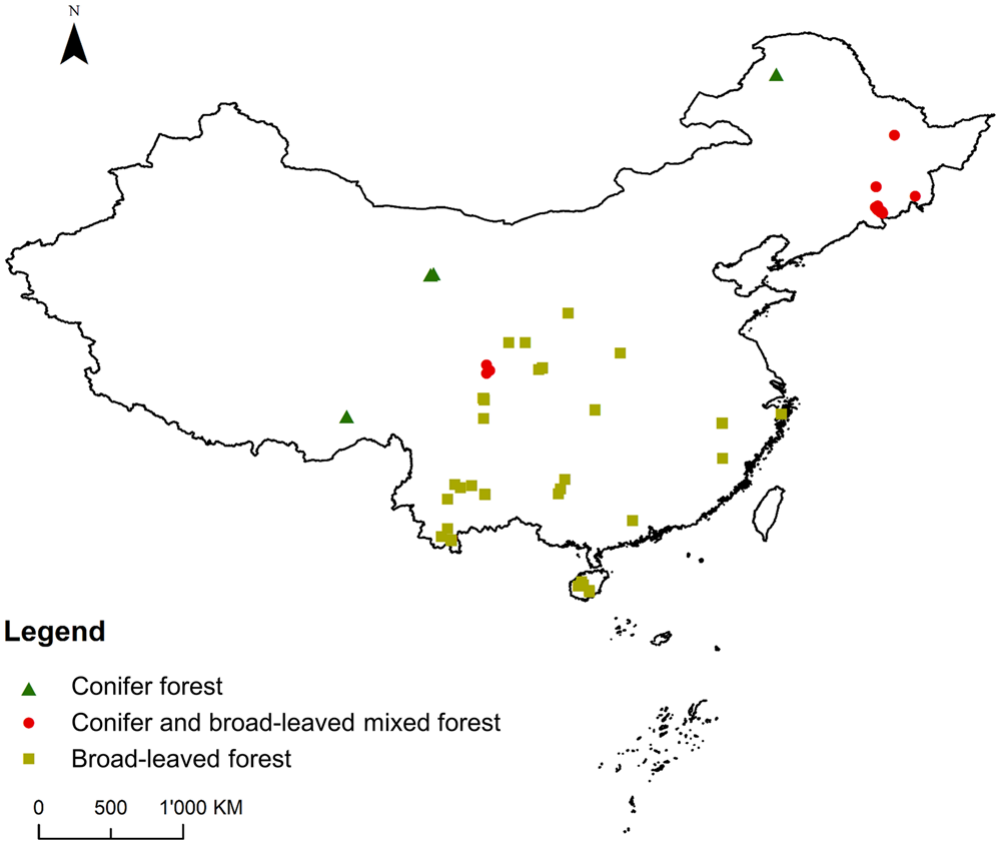
Study locations and forest types. This figure was created using ArcGIS by Esri (ArcMap10.5).

We included the 125 pairs of secondary-primary forest data across China (Fig. 1), which were collected in the previous meta-analysis^24^. The recovery was defined as the ratio of secondary forests woody plant species richness to the reference forests woody plant species richness, i.e. the primary forest woody plant species richness. Ecological drivers were indicated by the following variables: (i) logging type, (ii) recovery time after logging, (iii) annual temperature (°C), (iv) annual precipitation (mm/yr), (v) mean perimeter-area ratio of forest patches, (vi) proximity index, (vii) percentage of forest cover, (viii) regional species pool, and (ix) forest type.

In the regions where the secondary forests were collected, annual temperature ranged from −5 to 25 °C with a mean of 14.23 °C. Annual precipitation ranged from 250 to 2250 mm/yr with a mean of 1232.89 mm/yr. Since we only considered the secondary forests with presence of primary forests nearby (used as a reference for evaluating the recovery), the percentages of forest cover mainly varied between 60 to 100%. Regional species pools (woody plant species richness) ranged from 100 to 2200. The recovery time of these secondary forests mostly ranged from 5 to 120 years. The frequency histograms of these collected independent variables were shown in Supplementary Fig. S1.

To identify variables impacting the recovery ratio, models of all possible combinations of the variables of the global models were fitted and ranked based on their Akaike Information Criterion corrected for small sample sizes (AICc). The model with the lowest AICc included 8 variables and 6 interactions, the model ranked second had two fewer parameters, while the next most parsimonious model with 4 ∆AICc included only 5 variables and 1 interaction (Table 1). Given the small ∆AICc and the large difference in the number of parameters, the two more complex models are poor competitors of the simpler models, thus only the coefficient estimates of the latter model are presented here. The model included exclusively logging type, temperature, perimeter-area ratio, percentage of forest cover, regional species pool and the interaction between logging type and regional species pool. The relative importance of those variables (≥0.78) was shown in Table 2, and pseudo-R^2^ of this model was 0.42. Further support for this model was the rank based on Baysian Information Criteria (BIC). The BIC method more strongly penalizes for the number of parameters than the AIC. The model ranked first when using BIC, with a weight of 0.38. It had the lowest AICc in 3% of instances over 1,000 bootstraps and was the most parsimonious amongst the top 10 models with the highest percentages in the bootstrapping analysis.

**Table 1:** Candidate models for recovery ratios ranked according to ΔAICc, with their corresponding log likelihood (LogLik), degree of freedom (df) and Akaike weights (w).

Model rank	Variables	LogLik	df	ΔAICc	w
1	Logging type + Forest cover + Perimeter-area ratio + Regional species pool + Temperature + Forest type + Proximity + Precipitation + Logging type: Regional species pool + Logging type: Perimeter-area ratio + Logging type: Proximity + Logging type: Precipitation + Forest type: Precipitation + Forest type: Temperature	−16.43	19	0	0.02
2	Logging type + Forest cover + Regional species pool + Temperature + Forest type + Proximity + Precipitation + Logging type: Regional species pool + Logging type: Proximity + Logging type: Precipitation + Forest type: Precipitation + Forest type: Temperature	−19.46	17	0.54	0.02
7	Logging type + Forest cover + Perimeter-area ratio + Regional species pool + Temperature + Logging type: Regional species pool	−31.44	8	2.03	0.01

Only models with ΔAICc < 4 and fewer parameters were retained.

The symbol “:” means interaction.

**Table 2:** Relative importance of independent variables from AICc.

Independent variables and their interactions	Relative importance
**Logging type**	**1.00**
**Temperature**	**1.00**
**Forest cover**	**1.00**
**Regional species pool**	**1.00**
**Logging type: Regional species pool**	**0.99**
Forest type	0.92
Precipitation	0.85
**Perimeter-area ratio**	**0.78**
Proximity	0.74
Forest type: Precipitation	0.72
Logging type: Proximity	0.58
Logging type: Precipitation	0.52
Logging type: Perimeter-area ratio	0.43
Logging type: Temperature	0.42
Forest type:Temperature	0.42
Recovery time	0.32
Logging type: Forest cover	0.28
Forest type: Regional species pool	0.20
Forest type: Forest cover	0.14
Forest type: Proximity	0.12
Logging type: Recovery time	0.09
Forest type: Perimeter-area ratio	0.07
Forest type: Recovery time	0.05

The symbol “:” means interaction.

The variables included in the most parsimonious model within 4 ΔAICc are in bold.

**Figure 2:**
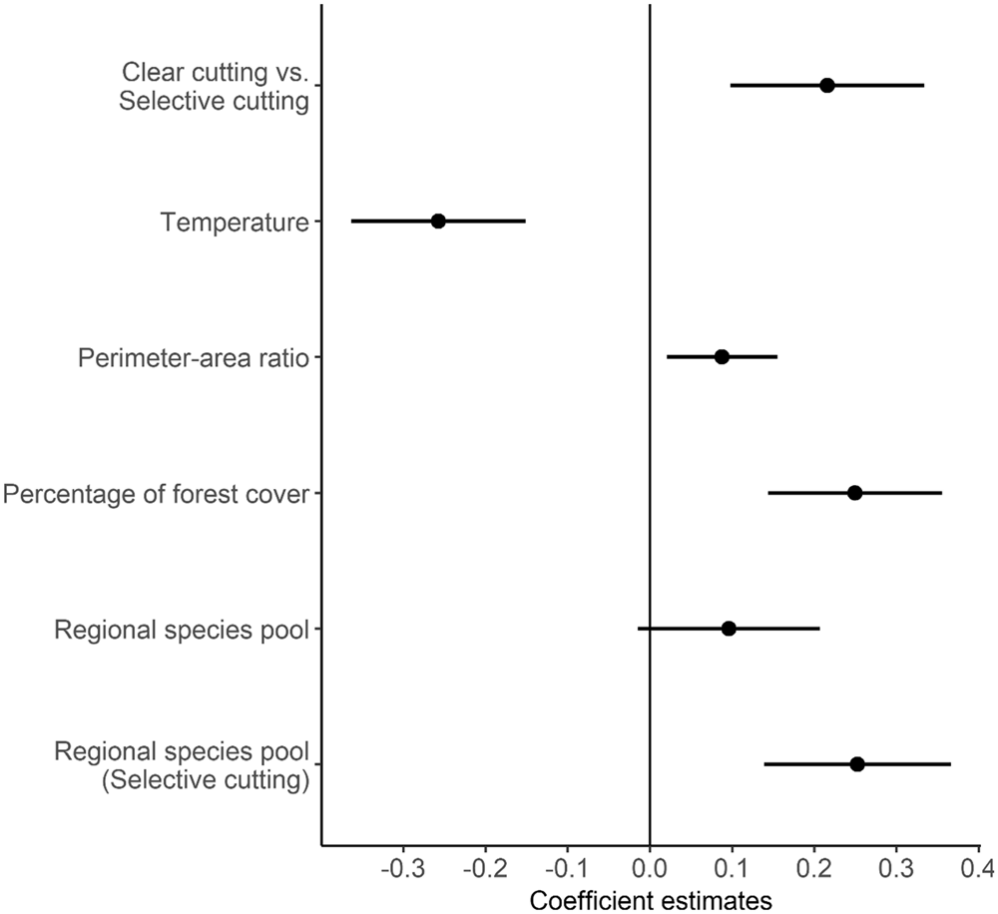
Variables and coefficient estimates for drivers of woody plant species richness recovery in secondary forests from the most parsimonious model within 4 ∆AICc. The coefficient estimates are for the natural log transformed recovery ratio and z-score rescaled continuous independent variables.

**Figure 3:**
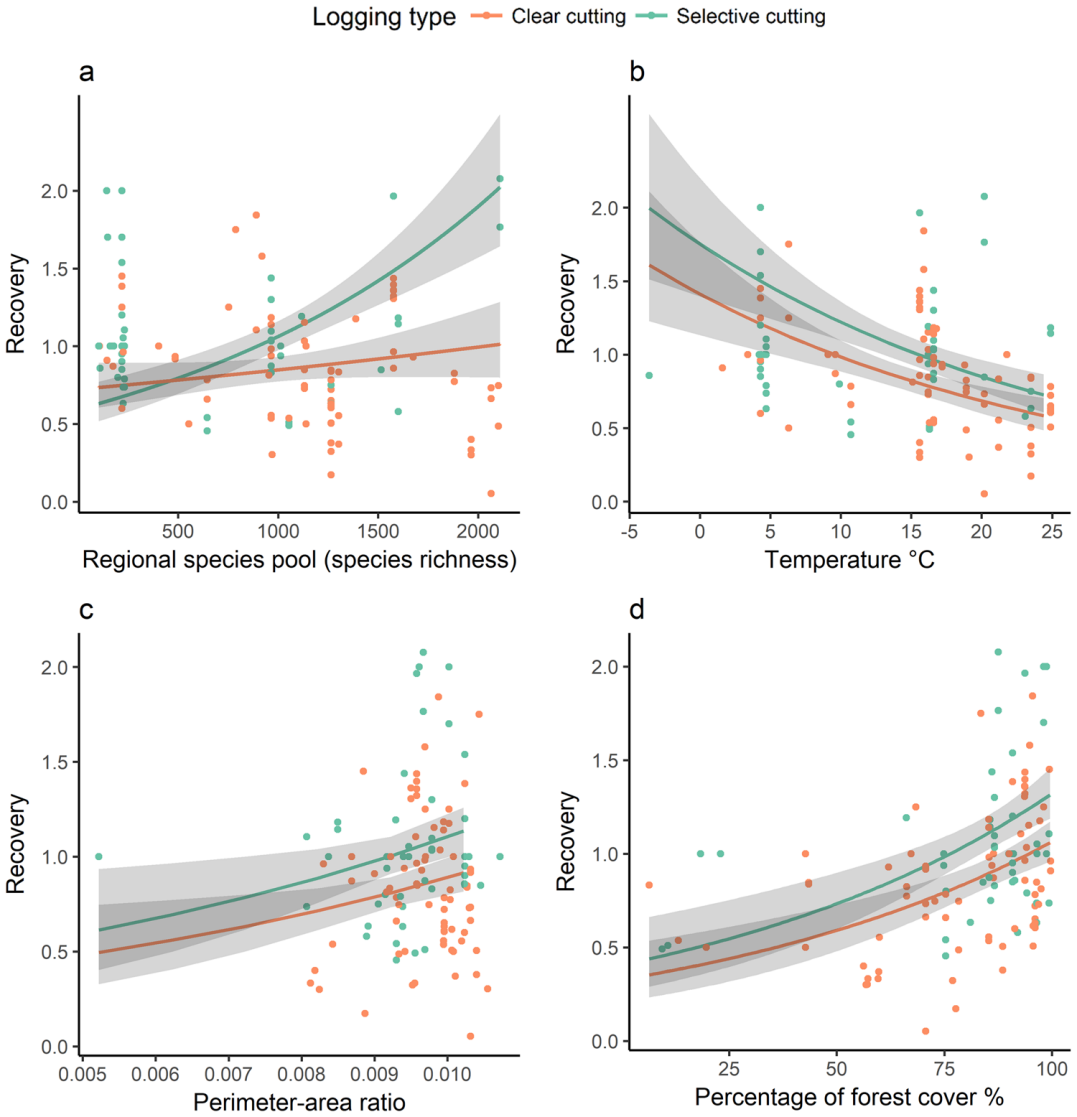
Relationships between the recovery of woody plant species richness and (**a**) regional species pool (**b**) temperature (**c**) perimeter-area ratio, and (**d**) percentage of forest cover (back-transformed). The pseudo-R^2^ of the most parsimonious model with the lowest AICc within 4 ∆AICc was 0.42. The dots are the observed data, the lines are the predictions from the model, and the shaded area is the 95% confidence interval.

The regional species pool was positively related to the recovery ratio of species richness and the slope of selective cutting forests was steeper than that of clear cutting forests (Figs 2 and 3a). The temperature was negatively related to the recovery ratios (Figs 2 and 3b). The perimeter-area ratio of forest patches (Figs 2 and 3c) and the percentage of forest cover (Figs 2 and 3d) had positive effects on the recovery ratios of species richness. The result was robust after removing the outlier for which perimeter-area ratio was 0.005 (Fig. 3c and Supplementary Table S1).

## Discussion

### Impacts of the regional species pool and logging type on the recovery of woody plant species richness

In regional species pool, the disturbance-adapted and disturbance-sensitive species can directly influence the ecological profile of plant recovery^9^. The observed positive relationship between the regional species pool and recovery ratio might be because the region with larger species pool contains more information legacies namely adaptations to historical disturbance cycles, and more material legacies such as soil seed bank^27^. Therefore it is more likely to have more disturbance-adapted species, which facilitate the seed availability^19^.

Additionally, there are multiple recovery trajectories of secondary forests^9,28^ because the recovery can be affected by various random factors and could reach different stable states other than the pre-disturbance state^29^. If the secondary forests would be recovered to pre-disturbance state, not only the regional species pool contains species that can unitedly play a leading role but also local dynamics are fast enough for early-arriving species to modify niches before other species arrive^30^.

This is partially proved by our results on logging type. Our results indicate that the effect of regional species pool on recovery is more pronounced in selective cutting forests than in clear cutting forests. In general, the selective cutting forests could recover better than clear cutting forests since the disturbance is lighter^22^ and there are more remnant forests^19^. In selective cutting forests, the niches have been basically fixed because of the existence of pioneer species and local early-arriving species, so the role of regional species pool in forest recovery can be very strong. However in the forests after clear cutting, at least in the early succession, the recovery is affected by the competition between local species and alien species, as well as other diverse random factors, thus the effect of regional species pool is less strong. Our study does not include the dynamic changes of species composition and if we can integrate the data of species composition, we can better explain the impact of regional species pool on species recovery.

Admittedly, recovery time is an indispensable factor when evaluating the forest regeneration^31–33^. However, our results did not show strong relationship between time and recovery ratio. This might be because our dataset include many different types of forest ecosystems. If the analysis is limited to a single forest ecosystem or in the same eco-region, we also expect that there would be a generalized linear correlation between recovery ratio and recovery time, as what has been found in other studies^31,34^. Furthermore, before the ecosystem stabilizing, a unimodal relationship is also possible since species richness can increase with pioneer species and then decrease because of competition during coexistence^35^. However, multiple ecosystems are included within the analysis, this pattern becomes less obvious. For example, the recovery pattern of broad-leaved forests in the south region is considerably different from that of conifer forests in the northeast region, where the regrowth rate of conifer forests in the northeast region is slower and the species richness of reference forests is lower.

### Impacts of the temperature on the recovery of woody plant species richness

Many previous studies have shown that plant species richness is high in the places with high temperature along the latitudinal gradient^36^. In contrast to this plant species richness distribution, we found that the recovery ratios of woody plant species richness would be high with low temperature. A study on Puerto Rico Island showed similar findings that relatively lower temperature and high precipitation facilitated forest recovery at high elevations^37,38^. Temperature could influence plants through physiological processes (e.g. evapotranspiration of plants is higher in warmer conditions), grow processes (e.g. lower daily minimum temperatures are supposed to accelerate growth rates in tropical forest trees^39^), soil nutrients (e.g. decreasing temperature is also associated with increasing soil C and N stocks^40^), and ecosystem resilience (e.g. resilience of tropical forest might be reduced at the higher temperature^41^). However the specific mechanisms of how temperature impacts recovery of plant species richness and plant diversity need to be further explored.

### Impacts of the landscape on the recovery of woody plant species richness

Our results suggest that higher forest cover could promote the species richness recovery. Indeed, surrounding forest are important sources for recolonization, especially for those forests with few soil seed banks^11^. As such, the landscape with higher forest cover is likely to contain more native species and remnants of primary forests^9^, which helps secondary forests recover to pre-disturbance states^7^. In landscapes with lower surrounding forest cover, the secondary forest may have a unique trajectory and is additionally influenced by its own biological and biotic factors^28,42^, particularly those affecting the reproduction and dispersal^9^. Our results also suggest that higher shape complexity of forest patches would be conducive to species richness recovery. This effect may be explained by positive effects of landscape heterogeneity on species richness in fragmented landscape^43^. Again, if we can further integrate data on species composition, in particular considering invasive species, it would help us to understand more deeply the impacts of landscape on plant species recovery.

## Conclusion

Following up on a previous meta-analysis on the patterns of species recovery acorss China^24^, we conducted a nationwide analysis of main drivers that influenced the recovery of woody plant species richness in secondary forests across the country. We collected the logging history, climate, forest landscape and forest attribute data and used an information-theory approach to identify the main drivers of species richness recovery in secondary forest. Results showed that forests with larger regional species pool had higher recovery ratios and the effect of regional species pool on recovery was stronger in selective cutting forests than that in clear cutting forests. We also found that the temperature was negatively related to the recovery. The shape complexity of forest patches and the percentage of forest cover in the landscape had positive effects on the recovery ratios of species richness. The results from our study provide more basic information on the natural recovery of secondary forests and deeper insight into resilience of secondary forests in China. We suggest future research to incorporate data on recovery of species composition, which can help to more accurately identify ecological drivers that affect recovery of plant diversity, as well as improve interpretation of these drivers on the forest recovery.

## Methods

### Ecological drivers and data collection

In this study, we considered four drivers of the recovery: logging history, climate, forest landscape and forest attribute. The logging history was indicated by (i) logging type (clear cutting or selective cutting) and (ii) recovery time after logging (in years, time since the last logging). The climate was indicated by (iii) annual temperature (°C) and (iv) annual precipitation (mm/yr). The forest landscape was indicated by (v) shape complexity of forest patches–measured as the mean perimeter-area ratio of forest patches, (vi) isolation–measured as the proximity index^44^, and (vii) dominance of forest cover in the landscape–measured as the percentage of forest cover. The forest attribute was presented by (viii) regional species pool–using woody plant species richness, and (ix) the forest type (conifer forests, conifer and broad-leaved mixed forests, and broad-leaved forests).

Logging type and recovery time for each data point were obtained from the previous meta-analysis^24^. Regional species pool of a forest site was defined as all the woody species occurring in the 50*50 km^2^ grid cell where the secondary forest was located and was obtained from the *Database of China’s Woody Plants*^36^. Since the regional species pool in our study was defined within a 50*50 km^2^ grid cell, we regarded it as one aspect of forest attribute. Annual temperature and precipitation were gathered from China Meteorological Data Service Center (http://data.cma.cn/). We used 300 m resolution land cover data for the year 2015 provided by the European Space Agency (http://maps.elie.ucl.ac.be/CCI/viewer/), which included eight forest cover classes. For the three landscape metrics, we used ArcGIS by Esri (ArcMap10.5) to generate 10 km radius buffers on the 300 m resolution land cover map, as a radius of 10 km buffer has been shown to be sufficient to conduct landscape analysis for plants communities^22^. To specify the location of secondary forest in each study, we tried to group sampling sites and extract the center location of sampling sites or the study area as the corresponding geographic coordinate, which means that the “landscape impact” was to reflect the unit of the analysis rather than single sampling site^22^. We contacted the authors to request this geographic information if the data were not available. Fragstats 4.2 was used to calculate the forest landscape metrics within each buffer area. The China administrative border map used in Fig. 1 was downloaded from http://www.resdc.cn/. The forest type (conifer forests, conifer and broad-leaved mixed forests, and broad-leaved forests) of each data point was judged according to both original literatures and eco-regions. We used the eco-region database defined by the WWF^45^.

### Data analysis

We fitted weighted generalized linear models (GLMs) and used an information-theory approach to identify the main drivers of species richness recovery in secondary forest^46^. The unit of analysis was a pair of secondary forests woody plant species richness (*SR*) and reference primary forests woody plant species richness (*PR*). In this study, the recovery ratio (*R*_*c*_) was calculated by Equation (1):

1 $${R}_{c}=\frac{SR}{PR}\,$$

Recovery ratio was natural log transformed and the continuous independent variables were rescaled using z-score. The weight of each pair of secondary-primary forest data reflect the precision and amount of information of a particular study in the meta-analysis^47^. We used the same weight as those used in the previous meta-anlysis^24^. We checked collinearity among drivers by assessing generalized variance inflation factor (GVIF) in each model and considered that a value of GVIF^1/(2*df)^^48^ above 10^(1/(2*df))^ indicated strong collinearity^49^ (df, degree of freedom). We did not detect strong collinearity in our dataset (Supplementary Table S2). We initially fitted one global weighted generalized linear models with the response variable recovery ratio and the nine potential independent variables listed above, as well as the interactions between logging type and all the continuous variables, and the interactions between forest type and all the continuous variables. However, such a model could not converge due to the high number of parameters, so instead, we fitted multiple global models each including one of all the possible combinations of 10 interactions. We then fitted all possible models involving at least one variables and rank them according to Akaike Information Criterion corrected for small sample sizes (AICc), with the MuMIn package^50^. We further used the Baysian Information Criteria (BIC) method to obtain more support for our results. As all model sets were performed on the same dataset, they were directly comparable. We reported only the model with the lowest AICc and all models within 4 ∆AICc units but with fewer parameters.

As the data collection procedure implied resampling in the study landscape, we controlled for pseudo-replication. We calculated the percentage of times a model was ranked with the lowest AICc after 1,000 bootstraps^22,51^. To speed up the computing process, bootstrapping was performed on a global model excluding all forest type interactions.

Finally, McFadden’s R square^52^, as one type of pseudo-R^2^ was calculated to show goodness of fit of the generalized linear models after model selection. Statistics analyses were conducted in R 3.4.1.

## Electronic supplementary material

Supplementary Information

Supplementary Dataset

## Data Availability

Data are available in the Supplementary Dataset.

## References

[1] Malhi Y, Gardner TA, Goldsmith GR, Silman MR, Zelazowski P (2014). Tropical Forests in the Anthropocene. Annu. Rev. Environ. Resour..

[2] Newton AC, Cantarello E (2015). Restoration of forest resilience: an achievable goal?. New Forests.

[3] Mace GM (2014). Whose conservation?. Science.

[4] Folke C (2006). Resilience: The emergence of a perspective for social–ecological systems analyses. Global Environ. Change.

[5] Poorter L (2016). Biomass resilience of Neotropical secondary forests. Nature.

[6] Barlow J (2007). Quantifying the biodiversity value of tropical primary, secondary, and plantation forests. Proc. Natl. Acad. Sci. USA.

[7] Norden N, Chazdon RL, Chao A, Jiang YH, Vílchez‐Alvarado B (2009). Resilience of tropical rain forests: tree community reassembly in secondary forests. Ecol. Lett..

[8] Moreno-Mateos D (2017). Anthropogenic ecosystem disturbance and the recovery debt. Nat. Commun..

[9] Arroyo-Rodriguez V (2017). Multiple successional pathways in human-modified tropical landscapes: new insights from forest succession, forest fragmentation and landscape ecology research. Biological Reviews.

[10] Brudvig LA (2017). Interpreting variation to advance predictive restoration science. J. Appl. Ecol..

[11] Sams MA (2017). Landscape context explains changes in the functional diversity of regenerating forests better than climate or species richness. Global Ecology and Biogeography.

[12] Holl KD, Reid JL, Chaves-Fallas JM, Oviedo-Brenes F, Zahawi RA (2017). Local tropical forest restoration strategies affect tree recruitment more strongly than does landscape forest cover. J. Appl. Ecol..

[13] Barbosa JM, Asner GP (2017). Prioritizing landscapes for restoration based on spatial patterns of ecosystem controls and plant-plant interactions. J. Appl. Ecol..

[14] Liu X (2015). The development of ecological impact assessment in China. Environ. Int..

[15] Xu WH (2017). Strengthening protected areas for biodiversity and ecosystem services in China. Proc. Natl. Acad. Sci. USA.

[16] Ren GP (2015). Effectiveness of China’s National Forest Protection Program and nature reserves. Conserv. Biol..

[17] Liu JG, Li SX, Ouyang ZY, Tam C, Chen XD (2008). Ecological and socioeconomic effects of China’s policies for ecosystem services. Proc. Natl. Acad. Sci. USA.

[18] Chazdon RL (2017). Landscape restoration, natural regeneration, and the forests of the future. Ann Mo Bot Gard.

[19] Chazdon, R. L. Second growth: the promise of tropical forest regeneration in an age of deforestation (University of Chicago Press, 2014).

[20] FAO. Global Forest Resources Assessment (FAO, 2015).

[21] Ren YJ, Lu YH, Fu BJ, Zhang K (2017). Biodiversity and ecosystem functional enhancement by forest restoration: a meta-analysis in China. Land Degrad. Dev..

[22] Crouzeilles R (2016). A global meta-analysis on the ecological drivers of forest restoration success. Nat. Commun..

[23] Newbold T (2014). A global model of the response of tropical and sub-tropical forest biodiversity to anthropogenic pressures. Proc. Royal Soc. B.

[24] Liu XF, Liu XH, Skidmore A, Garcia C (2017). Recovery of woody plant species richness in secondary forests in China: a meta-analysis. Sci. Rep..

[25] Gibson L (2011). Primary forests are irreplaceable for sustaining tropical biodiversity. Nature.

[26] Zobel M (2016). The species pool concept as a framework for studying patterns of plant diversity. J. Veg. Sci..

[27] Johnstone JF (2016). Changing disturbance regimes, ecological memory, and forest resilience. Front. Ecol. Environ..

[28] Stuble KL, Fick SE, Young TP (2017). Every restoration is unique: testing year effects and site effects as drivers of initial restoration trajectories. J. Appl. Ecol..

[29] Connell JH (1978). Diversity in tropical rain forests and coral reefs. Science.

[30] Fukami T (2015). Historical contingency in community assembly: integrating niches, species pools, and priority effects. Annu. Rev. Ecol. Evol. Syst..

[31] Liebsch D, Marques MC, Goldenberg R (2008). How long does the Atlantic Rain Forest take to recover after a disturbance? Changes in species composition and ecological features during secondary succession. Biol. Cons..

[32] Curran M, Hellweg S, Beck J (2014). Is there any empirical support for biodiversity offset policy?. Ecol. Appl..

[33] Laughlin DC (2017). The hierarchy of predictability in ecological restoration: are vegetation structure and functional diversity more predictable than community composition?. J. Appl. Ecol..

[34] Suganuma MS, Assis GBD, Durigan G (2014). Changes in plant species composition and functional traits along the successional trajectory of a restored patch of Atlantic Forest. Community Ecology.

[35] Chesson P (2000). Mechanisms of maintenance of species diversity. Annu. Rev. Ecol. Syst..

[36] Wang, Z., Fang, J., Tang, Z. & Lin, X. Patterns, determinants and models of woody plant diversity in China. *Proc. Royal Soc. B*., 10.1098/rspb.2010.1897 (2010). 10.1098/rspb.2010.1897 PMC3107620 21147804

[37] Crk T, Uriarte M, Corsi F, Flynn D (2009). Forest recovery in a tropical landscape: what is the relative importance of biophysical, socioeconomic, and landscape variables?. Landsc. Ecol..

[38] Daly C, Helmer EH, Quinones M (2003). Mapping the climate of Puerto Rico, Vieques and Culebra. Int. J. Climatol..

[39] Feeley KJ, Wright SJ, Supardi MNN, Kassim AR, Davies SJ (2007). Decelerating growth in tropical forest trees. Ecol. Lett..

[40] Tashi S, Singh B, Keitel C, Adams M (2016). Soil carbon and nitrogen stocks in forests along an altitudinal gradient in the eastern Himalayas and a meta-analysis of global data. Glob. Change Biol..

[41] Verbesselt J (2016). Remotely sensed resilience of tropical forests. Nat. Clim. Change.

[42] Fischer A, Marshall P, Camp A (2013). Disturbances in deciduous temperate forest ecosystems of the northern hemisphere: their effects on both recent and future forest development. Biodivers. Conserv..

[43] Redon M, Bergès L, Cordonnier T, Luque S (2014). Effects of increasing landscape heterogeneity on local plant species richness: how much is enough?. Landsc. ecol..

[44] Gustafson EJ, Parker GR (1992). Relationships between landcover proportion and indices of landscape spatial pattern. Landsc. Ecol..

[45] WWF. *Terrestrial ecoregions of the world*. https://www.worldwildlife.org/pages/conservation-science-data-and-tools# (2012).

[46] Burnham, K. P. & Anderson, D. R. Model selection and multimodel inference: a practical information-theoretic approach. (Springer Science & Business Media, 2003).

[47] Michael Borenstein, L. V. H., Julian P. T. H. & Hannah, R. Rothstein. *Introduction to Meta-Analysis* (John Wiley & Sons, 2009).

[48] Fox J, Monette G (1992). Generalized collinearity diagnostics. J Am Stat Assoc..

[49] O’Brien RM (2007). A caution regarding rules of thumb for variance inflation factors. Quality & Quantity.

[50] Barton, K. & Barton, M. K. Package ‘MuMIn’. Available at https://cran.r-project.org/package=MuMIn (2018)

[51] Davison, A. C. & Hinkley, D. V. *Bootstrap Methods and their Application* (Cambridge University Press, 2006).

[52] McFadden, D. Conditional logit analysis of qualitative choice behavior. In *Frontiers in Econometrics* (ed. P. Zarembka) 105–142 (Academic Press, 1974).

