# Supplementary material for: Main ecological drivers of woody plant species richness recovery in secondary forests in China
Source: Sci Rep. 2019 Jan 22;9:250. doi: 10.1038/s41598-018-35963-7 (PMC6342914; doi:10.1038/s41598-018-35963-7)
Supplement: Supplementary file 1 — Supplementary Information [file 41598_2018_35963_MOESM1_ESM.pdf]

Main ecological drivers of woody plant species richness recovery in secondary forests in China

Xiaofei Liu[1,2], John Garcia-Ulloa[2], Tina Cornioley[2], Xuehua Liu[1*], Zhiheng Wang[3] and Claude Garcia[2,4]

[1]State Key Joint Laboratory of Environmental Simulation and Pollution Control, and School of Environment, Tsinghua University, Beijing, 100084 China.

[2]Institute of Terrestrial Ecosystems, Department of Environmental Systems Science, Swiss Federal Institute of Technology Zurich (ETH Zurich), Zurich, 8092 Switzerland.

[3]Department of Ecology and Key Laboratory for Earth Surface Processes of the Ministry of Education, College of Urban and Environmental Sciences, Peking University, Beijing, 100871 China.

[4]Research Unit Forests and Societies, Centre International de Recherche Agronomique pour le Développement (CIRAD), Montpellier, 34392 France.

*Corresponding author: xuehua-hjx@mail.tsinghua.edu.cn

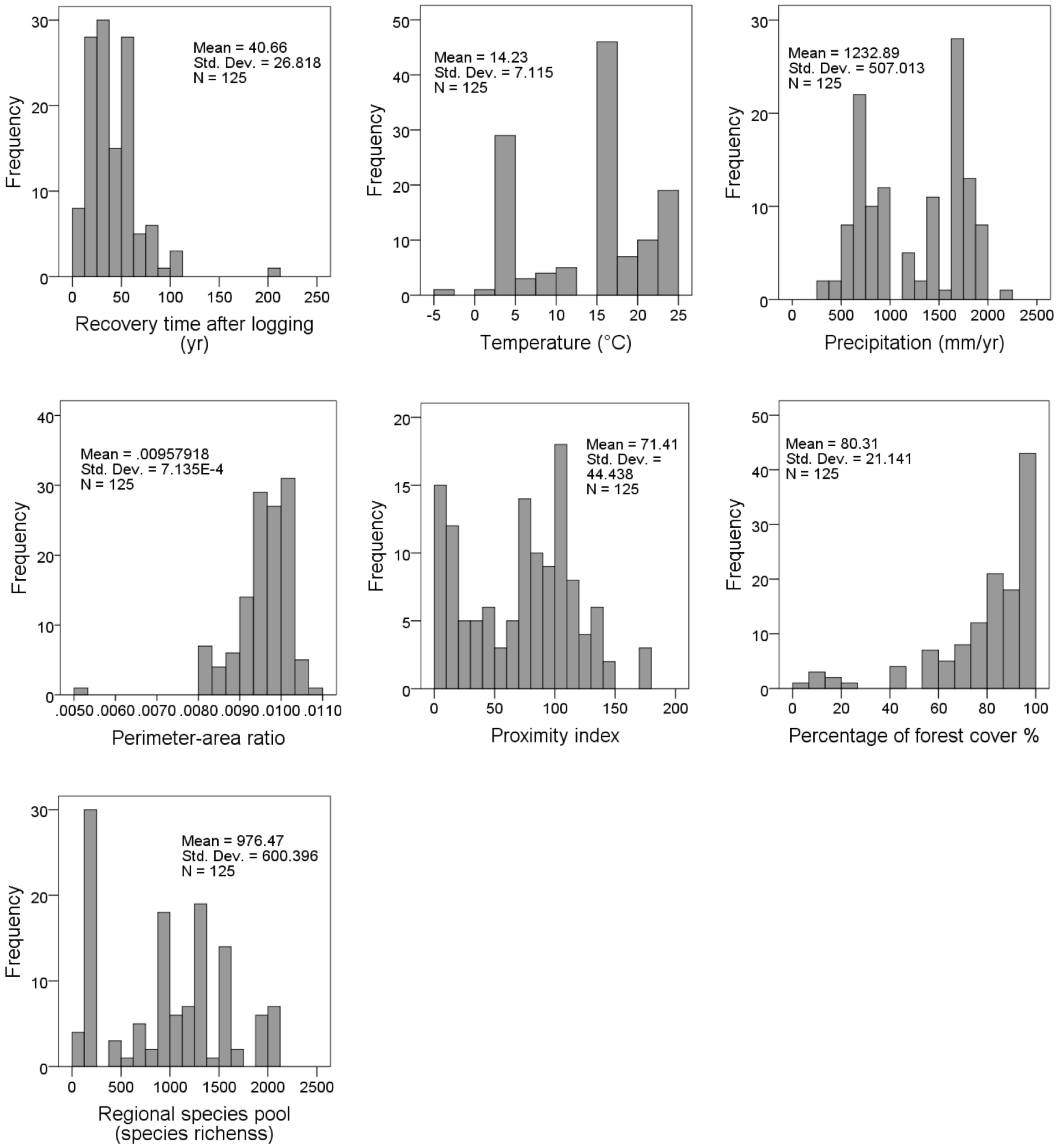

Figure S1. Frequency histograms of collected independent variables.

Table S1. Relative importance of independent variables from AICc without the outlier of Perimeter-area ratio

Independent variables	Relative importance
Logging type	1.00
Forest cover	1.00
Temperature	1.00
Regional species pool	0.98
Perimeter-area ratio	0.96
Logging type : Regional species pool	0.93
Forest type	0.56
Proximity	0.51
Precipitation	0.43
Logging type : Temperature	0.33
Logging type : Forest cover	0.32
Recovery time	0.29
Logging type : Proximity	0.27
Logging type : Perimeter-area ratio	0.25
Logging type : Precipitation	0.24
Logging type : Recovery time	0.07

The symbol ":" means interaction.

Table S2. Collinearity statistics of datasets (n=125)

Parameter	GVIF^(1/2*df)	df
Logging type	1.202	1
Forest type	1.568	2
Recovery time	1.101	1
Temperature	2.854	1
Precipitation	1.853	1
Perimeter-area ratio	1.074	1
Proximity	1.210	1
Forest cover	1.241	1
Regional species pool	2.030	1

Collinearity is strong when generalized variance inflation factor ($GVIF^{(1/(2*df))}$) is higher than $10^{(1/(2*df))}$[1,2]. df, degree of freedom.

References

1. Fox, J. & Monette, G. Generalized collinearity diagnostics. *J Am Stat Assoc.* **87**, 178-183 (1992).
2. O'Brien, R. M. A caution regarding rules of thumb for variance inflation factors. *Quality & Quantity* **41**, 673-690 (2007).
